# Students' Perspective on Undergraduate Research Experiences in Indonesian Dental Schools

**DOI:** 10.1155/2024/5898527

**Published:** 2024-05-11

**Authors:** Erik Idrus, Nieka A. Wahono, Rezon Yanuar, Yuniardini S. Wimardhani, Ria Puspitawati, Lisa R. Amir

**Affiliations:** ^1^Department of Oral Biology, Faculty of Dentistry, Universitas Indonesia, Jakarta 10430, Indonesia; ^2^Department of Pediatric Dentistry, Faculty of Dentistry, Universitas Indonesia, Jakarta 10430, Indonesia; ^3^Department of Pharmacology, School of Dentistry, Health Sciences University of Hokkaido, Ishikari-Tobetsu, Hokkaido, Japan; ^4^Department of Oral Medicine, Faculty of Dentistry, Universitas Indonesia, Jakarta, Indonesia; ^5^Dental Education Unit, Faculty of Dentistry, Universitas Indonesia, Jakarta 10430, Indonesia

## Abstract

Undergraduate (UG) research is considered as an essential part of dental education. Numerous dental schools have included required course-based undergraduate research in their curricula. However, the implementation of UG research courses in the curriculum may vary between dental schools. In the present study, we aimed to evaluate student perspectives on UG research in the curriculum of Indonesian dental schools. A total of 203 participants from 10 dental schools returned the questionnaire. The participants were clinical students of the dentistry profession program who completed their undergraduate dentistry program from 2017 to 2022. The majority of study participants favored UG research in the curriculum of the undergraduate dentistry study program. Less than 20% participants perceived UG research experiments were not important in dental education. Factors that influenced these perceptions included the availability of adequate time to complete the course and sufficient support from research supervisors. Recommendations for improvement included providing an adequate time to complete UG research and adequate supervision to guide students to understand the conceptual background information of the research topics, designs, and scientific communication of data interpretation. Regular monitoring of students' performance and progress would ensure completion of UG research courses in a timely manner. In conclusion, although UG research as a compulsory course in the Indonesian dental curriculum was well received by the students, overcoming the challenges is essential for the improvement of the research environment for undergraduate dental students.

## 1. Introduction

Higher education aims to give students the knowledge, competencies, and analytical thinking necessary to excel within their selected disciplines and make meaningful contributions to the broader community. It endeavors to broaden the intellectual perspectives of students, instill an enduring zeal for education, and nurture an ongoing commitment to personal and professional development [1, 2]. To achieve the educational goals, the university promotes active student participation in hands-on learning experiences, internships, and scholarly inquiries, which bridges the gap between theoretical understanding and its practical application [3, 4].

Dental education must be oriented toward the scientific method to ensure dental students are well-equipped to be able to critically assess new materials and methods of innovation in dentistry [5]. Their academic journey must be steeped in the scientific way to develop the scientific mindset, to ensure they are thoroughly prepared to deliver cutting-edge, evidence-based care to their patients upon entering the profession program [6, 7]. As technology in dentistry is rapidly evolving, dental professionals must remain current with emerging research and innovations. An education rooted in the scientific method will equip future dentists with the tools to assess new information critically, modify their practices as warranted, and contribute to ongoing progress within their field [6, 8, 9].

Engaging in undergraduate (UG) research is essential to a comprehensive higher education experience. These opportunities contribute to the improvement of dental students' academic knowledge as well as their critical thinking and problem-solving skills. By participating in UG research, students develop the ability to identify issues, devise solutions, and form conclusions based on empirical evidence. This exposure also familiarizes them with the standards and procedures of academic research, preparing them for advanced studies or a professional career in dentistry. Beyond the educational advantages, UG research also fosters a collaborative relationship between students and professors, allowing students to benefit from their mentors' extensive expertise and research experience, ultimately contributing to their professional development and expanding their network [10–12].

Comprehending the potential of UG research, dental schools in Indonesian universities have embedded it in their curriculums. Currently, there are 34 dental schools in Indonesia, all of which must follow the national curriculum standards regulated by the Indonesian Dentistry Collegium. UG research is included as one of the competencies of dental graduates. Each dental school provides opportunities, infrastructure, and resources to motivate students to undertake research projects. While its beneficial outputs are undebatable, UG research, for some dental students, might be very demanding and challenging [13–15]. Previously, we reported the perception of dental students and alumni of Universitas Indonesia on their experiences during UG research. The study presented evidence that UG research was well received with some recommendations for improvements. The recommendation included the autonomy to select research topics of interest and the opportunity to get involved in writing manuscripts with the supervisors [16]. Although the minimum Indonesian dentist competencies were regulated nationally, the arrangements and implementation of UG research may differ among dental schools. Therefore, the present study aimed to investigate the experience of implementing undergraduate research programs at dental schools in Indonesia. The research experiences and satisfaction and the impact on professional and academic careers for the students were also analyzed.

## 2. Material and Methods

### 2.1. Study Participants

The study was conducted from January to August 2023. In Indonesia, dental curriculum consists of 6 years of academic and professional (clinical) study program. Participants of the study were students who were on their clinical stage of the dentistry program. Convenience sampling techniques were applied. All the invited schools have implemented undergraduate research as part of their compulsory courses in the academic dentistry program. An invitation was sent to each schools' administration, and the participants gave their consent to participate in the study before filling out the online questionnaire. Ethical approval of this study was obtained from the Dental Research Ethics Committee (17/Ethical Approval/FKGUI/IV/2022).

### 2.2. Questionnaire

An online survey was developed with Google Forms to examine the perception of Indonesian dental students toward the undergraduate research program as one of the compulsory courses in their dental curriculum. The questionnaires were divided into four sections, which were general information, background information of undergraduate research, participants' perception regarding their preferences, learning experiences, learning satisfaction, and the impact of UG research on student academic performances. Overall, there were 29 questions in the questionnaire. Four Likert-type scales (1 = strongly disagree to 4 = strongly agree) were used for answering the perception section to avoid the neutral opinion. In addition, two open-ended questions that asked about students' challenges encountered during UG research and the students' recommendation for improvement were also included. These provided students with the opportunities to elaborate more on their perception. To lessen the acquiescence bias, the questionnaire was created with both positive and negative items. Reliability and validity test was performed as previously described [16]. Cronbach's alpha for the internal consistency reliability questionnaire was 0.908.

### 2.3. Statistical Analysis

Data distribution was analyzed with Kolmogorov–Smirnov test. Descriptive statistics and bivariate analyses were performed. Statistical significance was set at 0.05.

## 3. Results

The questionnaire was sent out to all dental schools in Indonesia. A total of 203 participants from 10 dental schools returned the questionnaire (response rate 29.4%). Participants of the study were students who were on their clinical stage of the dentistry program from 2017 to 2022. The majority of participants were from public dental schools. The characteristics of the study participants were described in Table 1.

This study showed that more than half of the participants conducted basic science studies for their UG research that involved both experimental or observational studies in the laboratorium. They responded that they had adequate time to perform the research. Most of the participants did not have experience presenting the results in a scientific conference nor participating in a scientific competition (Table 2).

Clinical students favored UG research as a compulsory course in the curriculum of the dentistry academic study program. Only less than 20% of participants agreed that UG research experiments were not important in dental education and should not be included as a compulsory course in the dental curriculum, as they were not satisfied with their experience of UG research (15.3%). While in the learning experience domain, more than 90% of participants agreed that the experience had taught them to think critically and independently (Table 3).

We further analyzed the correlation between negative preferences toward undergraduate research and the participants' profile. The negative preferences were that students were not satisfied with the experiences, and they believed the course was not important for dental students and should not be included as a compulsory course. The study demonstrated the negative preferences toward undergraduate research correlated with the allocated time to complete the course (Table 4).

Positive impact of UG research was perceived by the students. Particularly, the experiences benefited students in their education of professional programs, applying the theoretical knowledge and clinical practice and to eflecting the technological advancement of new materials in clinical practices (Table 5).

Based on the narrative comments in the open-ended questions, challenges during UG research included inadequate time to complete the course timely and difficulties in data analysis and data interpretation, particularly when the support from supervisors were lacking.

## 4. Discussion

UG research plays a pivotal role in shaping a comprehensive higher education journey. It does more than enrich academic understanding; it actively engages students in knowledge creation. When undergraduates undertake research, they are not just passive recipients of information, as is often the case in traditional classroom settings; instead, they become investigators. Designing experiments or methodologies teaches them the value of planning and structure. Moreover, as they gather and analyze data, they hone their analytic skills, learning to sift through information critically and to assess its significance effectively. Furthermore, UG research can open the door to the professional methodologies and ethical standards essential in research [17, 18].

The present study assessed students' perspectives of UG research experience from 10 dental schools, comprising both public and private dental schools across Indonesia. In general, dental students' perception of UG research from all dental schools was very positive, with more than 93% of respondents agreed that UG research benefits their academic success. Dental schools are fostering their students' interest in complexity, issues, and problem-solving through research, where students can learn new information, absorb it, and adjust to the changes in dentistry and practice that will inevitably occur. They also acquire transferable skills: critical thinking, collaboration, and communication, all of which serve them well in future careers [12, 19, 20].

In this study, 12.8% of students thought that UG research was not crucial in dental education, and 18.7% preferred that UG research was not a compulsory course in dental education. The results are generally in agreement with the data of a previous study, which examined the perception of dental students and alumni of the Universitas Indonesia (UI) that showed the majority agreed on the importance and benefit of UG research [16]. Nonetheless, the reasons for respondents had negative perspectives were different. In the UI study, the reasons for the unsatisfactory perspective of UG research were the absence of experiences in participating in scientific meetings, competition, or publication, while in the current study, there were no significant different perspectives on UG research among the students with or without such experiences. The negative perspectives toward UG research were primarily related to the duration of allocated time to finish the UG research. All the three negative perceptions on UG research (unsatisfactory experience, that UG research was not important, and UG research should not be compulsory) were primarily shown by those with no adequate time to complete the course. The limited time provided to complete the course most likely did not give adequate opportunity for students to absorb and understand the essence of the research in a critical and analytical way of thinking so that they could not reach the satisfaction and the importance of doing the course. Such a result is in line with previous study with respondents of science, technology, engineering, and mathematics students who were exposed to undergraduate research experiences. The longer the duration of research experience exposure, the stronger the positive perspective of the students toward UG research [21]. Another previous study in Iran confirmed that research duration was a significant institutional barrier for UG students in performing UG research [22]. Time constraint was one of the obstacles in performing UG research, which was associated with the research time frame and preparing the research manuscript to be published in a high-impact journal [23–26]. The students may be concerned about whether their results are proper for publication; thus, they spend more time pushing their limits to produce “good results.” This study, however, did not determine the reason for inadequate time to conduct UG research. Therefore, a comprehensive strategy should be carefully developed as research duration was associated with the occurrence of student depression in the United States [23].

Research supervisors play a crucial role in assisting students to develop scientific skills, such as constructing an argument from evidence, and to understand the conceptual framework and research background. Students often struggle to interpret the findings, relate analyses to research objectives, or comprehend the reasoning behind the experimental design. Supervisors have an obligation to guide students to analyze the data and their interpretation. A good supervisor must be supportive and empowering, apply direct learning, and build a personal rapport by developing a similar style and interests with their supervised students. Inadequate supervision to discuss the research projects may lead to negative perceptions toward the UG research [16]. Therefore, the supervisors must have adequate resources and training before they supervise the UG research students [27, 28].

Studies conducted on multinational dental students revealed that a low percentage of students chose researchers as their future career and found researchers unattractive [29, 30]. This condition is becoming more concerning, considering the decreasing number of dental researchers based on previous reports [31]. Dental schools are the main source of dental researchers, and they hold enormous responsibilities to create future dental researchers. There must be an immediate strategy to change students' perspectives toward dental researchers as their future career. One of the strategies is exposing undergraduate dental students to a research atmosphere by conducting their own research project. UG research would prepare them to become dental researchers who would be involved in the creation of novel inventions to improve oral health. Our present study revealed the positive perception of Indonesian dental students toward UG research. This result indicates a positive sign and might reflect their interest in becoming future dental researchers.

### 4.1. Study Limitation

Our findings present evidence of the positive impact of undergraduate research as a compulsory course in the dental curriculum of the dentistry study program. However, the study has several limitations. The first is the low response rate, as the data obtained from 10 Indonesian dental schools only represent 24.9% of the total population. We distributed the questionnaire to 10 Indonesian dental schools through the executive head of the faculties and could not directly monitor the distribution process to their students. A number of suggestions to tackle the issue of low response rate in the future studies include conducting joint research collaboration with participating dental schools for better monitoring and direct distribution of the questionnaire. Furthermore, the responses from dentists on the effect in their professional careers could add more depth on understanding the impact of UG research. The perspective of research supervisors on the implementation of UG research as a compulsory course in their dental curriculum is another important factor for the overall evaluation.

## 5. Conclusions

The study presents evidence that Indonesian dental students have responded favorably to UG research as a required course in the curriculum which could assist in the development of future dentists with a scientific mindset. Future dentists can benefit from adopting this approach as it helps to shape their analytical thinking, which will help them process, evaluate, and integrate the data they collect from their clinical practice into the research community. Among the recommendations for improvement for the study were the provision of sufficient allocated time for UG research course and sufficient supervision to help students comprehend the conceptual background knowledge of the research topics, designs, data analysis, and interpretation. Developing comprehensive strategies to overcome the challenges is essential for the improvement of the research environment for undergraduate dental students.

## Figures and Tables

**Table 1:** General information of the dental schools and the study participants.

General information	Total (%)
1. Type of university	
a. Public	7 (70)
b. Private	3 (30)
2. Gender	
a. Male	40 (19.70)
b. Female	163 (80.30)
3. Average GPA	3.27 ± 0.24
4. Year of graduation of the bachelor program	
a. 2017	9 (4.43)
b. 2018	6 (2.96)
c. 2019	13 (6.40)
d. 2020	29 (14.29)
e. 2021	85 (41.87)
f. 2022	61 (30.05)

**Table 2:** The characteristics of the undergraduate research.

Undergraduate research	*N* (%)
1. Duration of undergraduate research	
a. Less than 3 months	16 (7.88)
b. 3–6 months	82 (40.39)
c. More than 6 months	105 (51.72)
2. There is adequate time to complete the course	
a. Not adequate	21 (10.34)
b. Adequate	16 (7.88)
c. More than adequate	166 (81.77)
3. Research expenses	
a. No expenses	27 (13.30)
b. Self-funded	160 (78.82)
c. Funding from supervisor	6 (2.96)
d. Funding form faculty	6 (2.96)
e. Partially funded	4 (1.97)
4. Undergraduate research was beneficial for my academic success and professional career	
a. Yes	190 (93.60%)
b. No	13 (6.40%)
5. Research topics	
a. Basic science studies	105 (51.72%)
b. Epidemiological studies	49 (24.14%)
c. Clinical studies	49 (24.14%)
6. Research supervisors	
a. Professors	14 (6.90%)
b. Lecturers (PhD)	47 (23.15%)
c. Lecturers (master degree)	57 (28.08%)
d. Lecturers (specialization degree)	85 (41.87%)
7. Presentation in scientific conferences	
a. International conferences	5 (2.46%)
b. National conferences	1 (0.49%)
c. Local conference	7 (3.45%)
d. No experience	190 (93.60%)
8. Participation in scientific competition	
a. International event	8 (3.94%)
b. National event	7 (3.45%)
c. Local event	—
d. No experience	188 (92.61%)
9. Publication of UG research	
a. Published in International journals	9 (4.43%)
b. Published in national journals	22 (10.84%)
c. Manuscript in preparation	42 (20.69%)
d. Store in repositories	96 (47.29%)
e. Not published	34 (16.75%)

**Table 3:** Indonesian dental students' perception on the experiences of UG research.

Statements on undergraduate (UG) research	Likert score				
	Strongly disagree	Disagree	Agree	Strongly agree	Domain mean ± SD
A. Preference domain	—	—	—	—	1.86 ± 0.75
1. UG research is not important for academic dental education	71 (34.98%)	106 (52.22%)	19 (9.36%)	7 (3.45%)	—
2. UG research preferably not included as compulsory course in dental curriculum	61 (30.05%)	104 (51.23%)	32 (15.76%)	6 (2.96%)	—
B. Learning experiences domain	—	—	—	—	3.18 ± 0.56
3. UG research improved my problem solving skills	2 (0.99%)	10 (4.93%)	140 (68.97%)	51 (25.12%)	—
4. UG research taught me to think independently	2 (0.99%)	4 (1.97%)	149 (73.40%)	48 (23.65%)	—
5. UG research improved critical thinking skills	2 (0.99%)	6 (2.96%)	135 (66.50%)	60 (29.56%)	—
6. Improve my ability to deliver presentation in seminars	1 (0.49%)	18 (8.87%)	136 (67.00%)	48 (23.65%)	—
7. UG research thought me the relation of research and clinical practice	3 (1.48%)	15 (7.39%)	138 (67.98%)	47 (23.15%)	—
C. Learning satisfaction domain	—	—	—	—	2.73 ± 0.80
8. UG research experience during my academic study was not satisfactory	52 (25.62%)	120 (59.11%)	27 (13.30%)	4 (1.97%)	—
9. UG research experience quite satisfactory	3 (1.48%)	16 (7.88%)	160 (78.82%)	24 (11.82%)	—
10. UG research experience was supported by adequate research facilities	11 (5.42%)	41 (20.20%)	128 (63.05%)	23 (11.33%)	—
11. UG research experience have adequate supports from supervisors	5 (2.46%)	14 (6.90%)	125 (61.58%)	59 (29.06%)	—

**Table 4:** Correlation of negative preference toward undergraduate research with researchers profile.

Variables	UG research experience during my academic study was not satisfactory		UG research is not important for academic dental education		UG research preferably not included as compulsory course in dental curriculum	
	Mean (SD)	*p*	Mean (SD)	*p*	Mean (SD)	*p*
University						
a. Public	1.88 (0.67)	0.313	1.81 (0.74)	0.881	1.89 (0.75)	0.305
b. Private	2.00 (0.70)		1.81 (0.72)		1.97 (0.77)	
Graduation year						
a. 2019–2020 (before the COVID-19 pandemic)	1.92 (0.75)	0.972	1.84 (0.84)	0.944	1.92 (0.79)	0.943
b. 2021–2020 (during the COVID-19 pandemic)	1.91 (0.65)		1.80 (0.70)		1.91 (0.74)	
Duration of UG research						
a. Less than 3 months	1.87 (0.71)	0.148	1.75 (0.77)	0.870	1.81 (1.79)	0.029^*∗*^
b. 3–6 months	1.85 (0.75)		1.73 (0.77)		1.79 (0.73)	
c. More than 6 months	1.91 (0.68)		1.88 (0.71)		2.02 (0.75)	
There is adequate time to complete the course						
a. Not adequate	2.19 (0.51)	0.013^*∗*^	2.09 (0.70)	0.041^*∗*^	2.23 (0.70)	0.025^*∗*^
b. Adequate	1.89 (0.67)		1.78 (0.73)		1.89 (0.75)	
c. More than adequate	1.75 (0.85)		1.75 (0.72)		1.91 (0.75)	
Presentation in scientific conferences						
International	1.80 (0.44)	0.226	1.60 (0.54)	0.866	1.80 (0.44)	0.561
National	1.00 (0)		1 (0)		1 (0)	
Local	1.71 (0.75)		2.28 (1.38)		2.00 (1.15)	
No	1.93 (0.68)		1.80 (0.71)		1.92 (0.74)	
Participation in scientific competition						
International	1.75 (0.46)	0.146	1.62 (0.51)	0.273	1.62 (0.74)	0.305
National	1.57 (0.78)		1.57 (0.78)		1.85 (0.89)	
Local	0.00 (0.00)		0.00 (0.00)		0.00 (0.00)	
No	1.93 (0.68)		1.82 (0.71)		1.93 (0.75)	
Publication of UG research						
International	1.66 (0.50)	0.109	1.77 (0.66)	0.757	1.77 (0.97)	0.101
National	2 (0.75)		1.77 (0.75)		1.95 (0.78)	
Manuscript	1.78 (0.64)		1.88 (0.86)		1.71 (0.80)	
Repository	1.91 (0.69)		1.77 (0.70)		1.98 (0.73)	
No	2.08 (0.66)		1.88 (0.72)		1.91 (0.75)	

^*∗*^*p* < 0.05.

**Table 5:** Indonesian dental students' perception on the impact of UG research.

Statements on undergraduate (UG) research	Likert score				
	Strongly disagree	Disagree	Agree	Strongly agree	Domain mean preference ± SD
D. Impact of UG research	—	—	—	—	3.02 ± 0.65
1. Helps me to apply my theoretical knowledge and clinical practice	5 (2.46%)	13 (6.40%)	148 (72.91%)	37 (18.23%)	—
2. UG research stimulate me to pursue academic careers	6 (2.96%)	42 (20.69%)	116 (57.14%)	39 (19.21%)	—
3. Help me to reflect technological advancement of new dental materials in clinical practice	3 (1.48%)	19 (9.36%)	135 (66.50%)	46 (22.66%)	—
4. Motivate me to continue postgraduate education	7 (2.3%)	86 (27.7%)	64 (20.6%)	153 (49.4%)	—

## Data Availability

Data are available upon reasonable request.
